# Endoscopy-assisted intrathecal morphine pump implantation for severe cancer pain: a case report

**DOI:** 10.3389/fonc.2026.1618305

**Published:** 2026-04-24

**Authors:** Yun Tong, Lanying Yu, Kaifeng Luo, Xiong Yan, Qinqin Hu, Liusheng Hu, Yihong Li, Libin Wang

**Affiliations:** 1 Department of Pain, The Affiliated Hospital of Jiujiang University, Jiujiang, China; 2 Hospital Infection Management Department, The Affiliated Hospital of Jiujiang University, Jiujiang, China

**Keywords:** cancer pain, endoscopic-assisted surgery, epidural fibrosis, intrathecal drug delivery system, refractory pain

## Abstract

This article presents a successful case of endoscopic-assisted intrathecal morphine pump implantation for the treatment of cancer pain associated with bone metastases. The patient was a 45-year-old female with severe cancer pain resulting from multiple bone metastases of rectal malignancy, which was poorly managed by the traditional three-step analgesic ladder. Preoperative intrathecal test puncture was attempted at multiple spinal levels but failed to access the intrathecal space. Subsequently, an endoscopic-assisted intrathecal morphine pump implantation was successfully performed at the L5-S1 level. Postoperatively, the patient achieved marked and sustained pain relief, accompanied by a dramatic improvement in her quality of life. No significant complications were observed during a 6-month follow-up. This technique provides a new therapeutic option for cancer pain patients who encounter difficulties with standard pump implantation procedures, demonstrating considerable potential for clinical application.

## 1 Introduction

Chronic cancer-related pain (CCRP) poses a significant global health challenge due to its high prevalence and detrimental impact on patient quality of life (1, 2). Pain associated with bone metastases is particularly complex and severe (3). Despite adherence to established analgesic guidelines, including the World Health Organization (WHO) three-step analgesic ladder, a substantial proportion of patients—estimated at 10% to 30%—experience refractory pain with inadequate relief or intolerable side effects from systemic pharmacotherapy (4).

For these patients with refractory cancer pain, implantable intrathecal drug delivery systems (IDDS) are a well-established and evidence-supported therapeutic option, offering superior pain control with reduced systemic side effects compared to conventional approaches (5, 6). However, successful implantation of an intrathecal catheter via standard percutaneous puncture can be exceptionally challenging or even impossible in patients with altered spinal anatomy.

These anatomical barriers often necessitate conversion to more invasive open surgical techniques for catheter placement, thereby increasing procedural risk, complexity, and patient burden. Herein, we present a technical case report of a successful intrathecal morphine pump implantation in a patient with severe refractory pain from rectal cancer bone metastases, in whom standard percutaneous access failed. We describe the utilization of endoscopic assistance to overcome the anatomical obstacles and achieve precise catheter placement. This technique may represent a valuable minimally invasive alternative for patients facing similar implantation challenges.

## 2 Case description

The patient, a 45-year-old female, presented with a two-year history of generalized pain. Two years prior, she had been diagnosed at an external hospital with rectal adenocarcinoma and multiple secondary bone metastases, which were accompanied by lumbosacral and bilateral lower limb pain. She underwent three courses of radiotherapy and chemotherapy, yet the disease showed persistent progression. Analgesic treatment at the previous hospital included oral oxycodone hydrochloride extended-release tablets (10 mg, q12h). Due to inadequate pain control, pregabalin capsules and flurbiprofen axetil were added, but the pain remained poorly managed. Severe nausea and vomiting developed. The oxycodone dose was progressively increased to 520 mg, q8h, with unsatisfactory effect, leading to her admission to our department.

The patient experienced severe postprandial vomiting. Considering her diagnosis of multiple secondary malignant tumors, refractory pain, and the severe adverse effects (nausea/vomiting) preventing further opioid escalation, with an estimated survival of 2–10 months, a semi-implantable intrathecal drug (morphine) infusion system implantation was planned after multidisciplinary discussion.

Her past medical history includes hypertension, rectal malignant tumor, and multiple bone metastases. She denied a history of diabetes, smoking, or alcohol use.

Physical examination revealed an ill-appearing, conscious patient with spinal scoliosis. There was significant tenderness over multiple lumbar spinous processes and interspaces, as well as over bilateral sciatic nerve points. The straight leg raise test was negative bilaterally. Muscle strength and tone in the lower extremities were normal, with no sensory deficits. Patellar and Achilles tendon reflexes were normal. During pain episodes, her Numeric Rating Scale (NRS) score was 9-10, with 5–8 episodes of breakthrough pain during the daytime.

The key clinical events during her hospitalization for the implantation procedure and the subsequent follow-up are summarized in **Table 1**.

**Table 1:** Timeline of key clinical events during hospitalization and follow-up for endoscopy-assisted intrathecal pump implantation.

Time point	Event description
Preoperative	Preoperative assessment (including lumbar MRI); multidisciplinary decision for intrathecal morphine pump implantation
Day of surgery	Failed fluoroscopy-guided catheter placement; successful endoscopy-assisted intrathecal catheter placement and pump implantation at L5–S1.
Postoperative Day 0	Initial bolus: 4 mg morphine; continuous infusion started at 0.166 mg/h.
Postoperative Day 1	Regular assessment; dose titration based on NRS and breakthrough pain.
Postoperative Day 2	Dosage adjusted to 1.8 mg/h (0.4 mg/ml concentration).
Postoperative Day 3	Pain stabilized (NRS 1–3); BTP reduced to 1–2 episodes/day.
Postoperative Day 4	Discharge with community-based pump management plan.
1 Month Post-op	NRS 1–2; reduced breakthrough pain; improved mobility.
3 Months Post-op	NRS 1–2; minimal breakthrough pain; independent daily activities.
6 Months Post-op	NRS 1–2; no significant breakthrough pain; normal activities resumed.

## 3 Diagnosis, therapeutic intervention, follow-up, and outcomes

### 3.1 Diagnosis and evaluation

**Figure 1:**
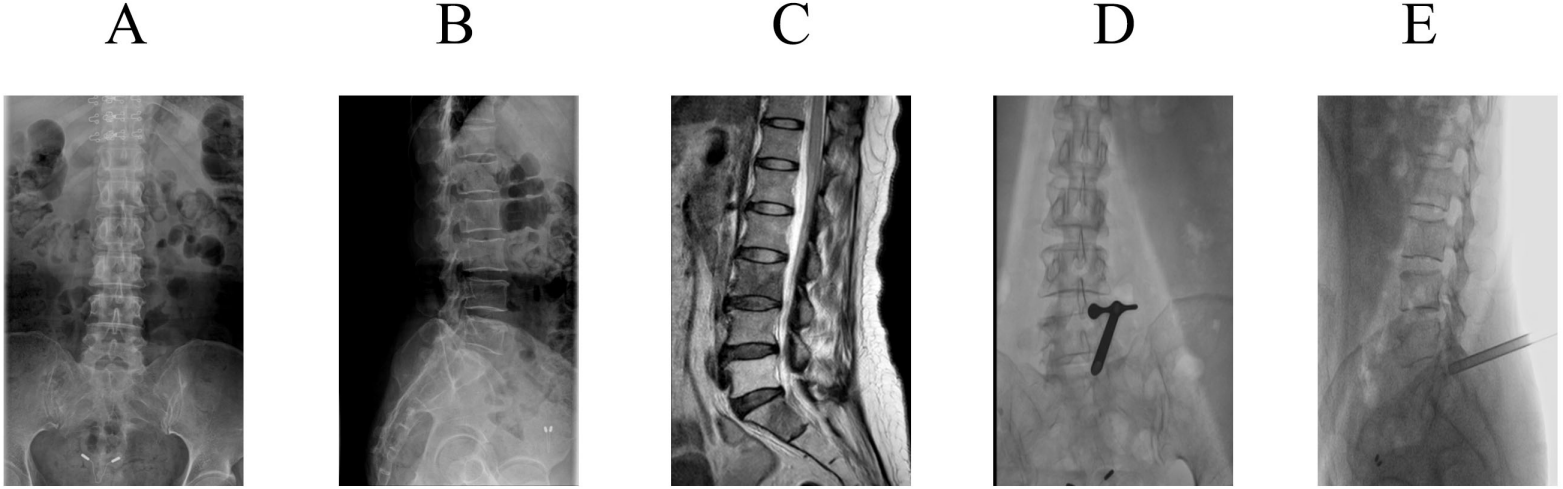
Preoperative and intraoperative imaging of the patient. **(A)** Anteroposterior (AP) lumbar spine digital radiograph. **(B)** Lateral lumbar spine digital radiograph. **(C)** Preoperative magnetic resonance imaging (MRI) of the lumbar spine showing bone metastasis. **(D)** Anteroposterior fluoroscopic image demonstrating the positioning of the endoscopic working cannula. **(E)** Lateral fluoroscopic image demonstrating the positioning of the endoscopic working cannula.

The patient suffered from severe refractory cancer pain secondary to multiple bone metastases from rectal carcinoma. She met the standard indications for implantable intrathecal drug delivery system (IDDS) therapy, including failure of comprehensive medical management and intolerable side effects from systemic opioids, as per established consensus guidelines (5). Preoperative magnetic resonance imaging (MRI) not only revealed metastatic involvement of the third lumbar vertebra but, more critically for the procedure, demonstrated extensive epidural fibrosis and scarring at the L4-L5 and L5-S1 levels, with obscuration of the normal interlaminar anatomical landmarks (**Figures 1A–C**). This finding prospectively indicated a high likelihood of failure for standard blind percutaneous puncture. Her pain was severe, with a Numeric Rating Scale (NRS) score of 9–10 and frequent daily episodes of breakthrough pain.

### 3.2 Therapeutic intervention

Prior to surgery, fluoroscopically-guided percutaneous puncture was attempted at the L2-L3 and L3-L4 interspaces, which are conventional access points for intrathecal catheter placement. However, these attempts failed to access the intrathecal space due to the obscured anatomy. The L4–L5 interspace was then selected but also failed. Consequently, the procedure was converted to the pre-planned endoscopic-assisted approach at the L5–S1 level.

The endoscopic-assisted procedure was performed as follows: The patient was positioned prone with an abdominal cushion. After standard skin preparation, local anesthesia was administered. A 16G puncture needle was advanced to the ligamentum flavum at the L5–S1 interspace under fluoroscopy. A guidewire was inserted, followed by sequential dilation and placement of a working cannula (**Figures 1D, E**). The transforaminal endoscope was then introduced.

**Figure 2:**
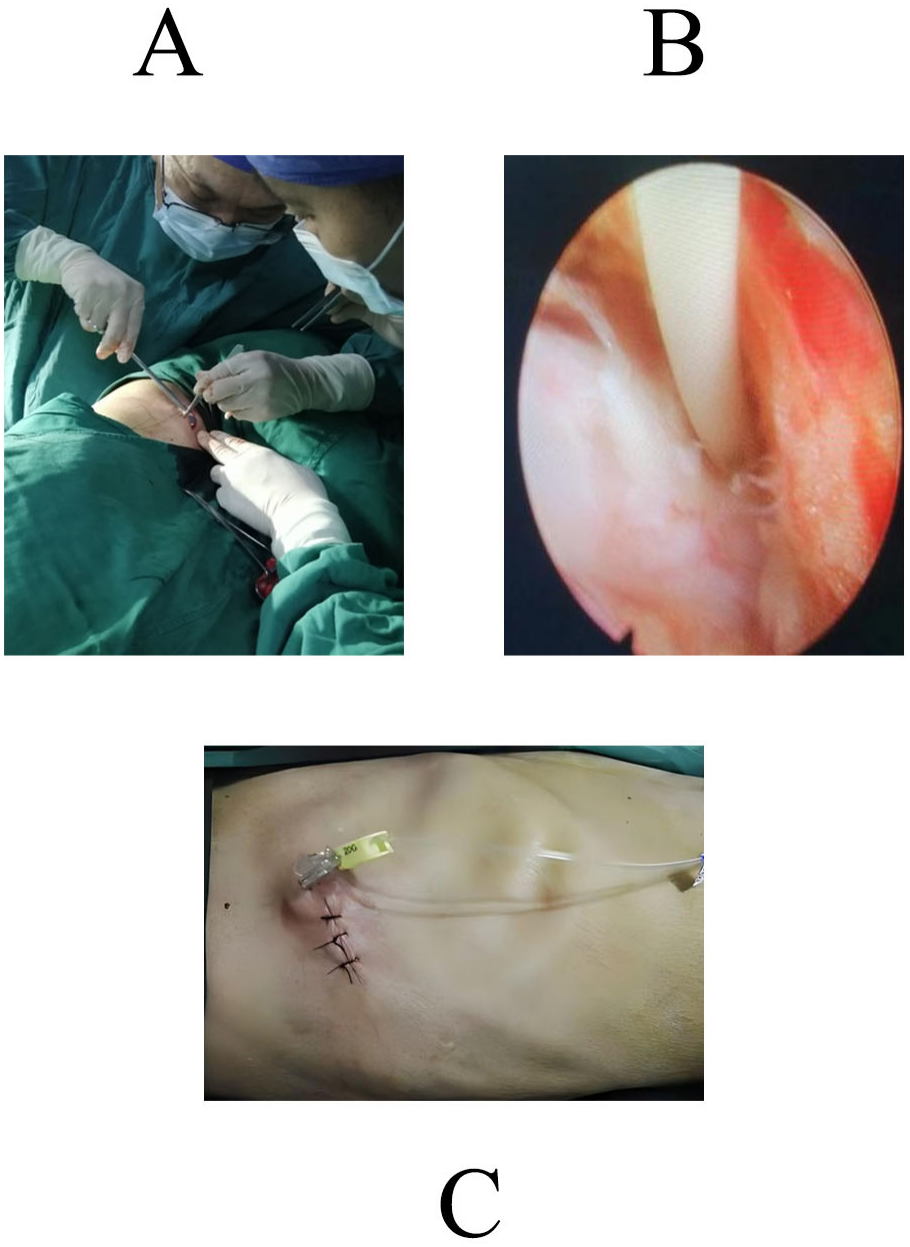
Intraoperative and postoperative photographs of the patient. **(A)** The distal end of the catheter was tunneled subcutaneously to the right midaxillary line at approximately the level of the 9th rib, where it was connected and secured to the implantable morphine pump, which was then placed subcutaneously; the incision was closed and sutured. **(B)** The dedicated silicone catheter for intrathecal pump implantation. **(C)** Postoperative incision site showing the subcutaneous fixation of the morphine pump and the external morphine liquid connection device.

Under direct endoscopic vision, the ligamentum flavum was identified as a yellowish, dense structure and incised to expose the dura mater. Hemostasis was achieved with a radiofrequency probe. Under endoscopic guidance, a 16G needle was used to access the intrathecal space, with free flow of cerebrospinal fluid (CSF) confirming correct placement. A dedicated silicone intrathecal catheter (**Figure 2A**) was inserted. The catheter was tunneled subcutaneously to the right flank and connected to an implantable morphine pump (Model 8637-20, Medtronic, USA), which was placed in a subcutaneous pocket (**Figure 2B**). Incisions were closed in layers (**Figure 2C**).

### 3.3 Follow-up and results

**Figure 3:**
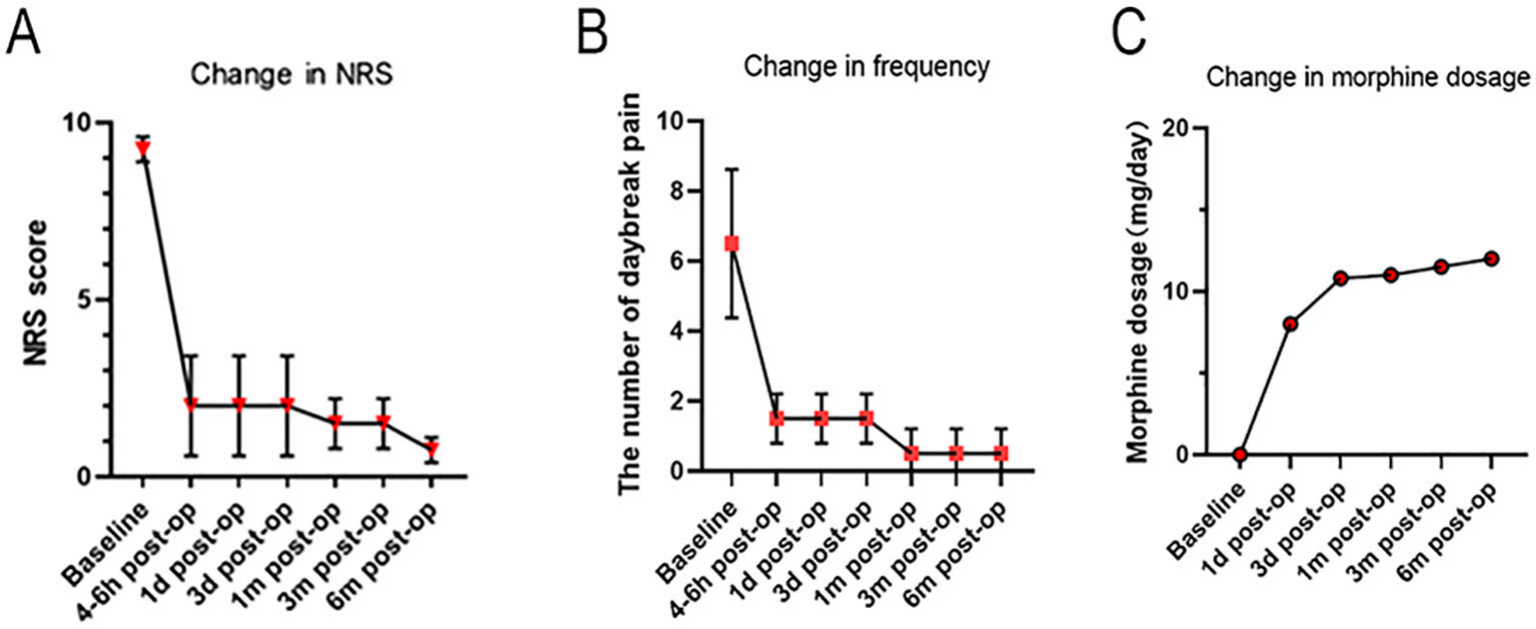
Postoperative morphine dosage and pain follow-up scores. **(A)** Changes in the Numeric Rating Scale (NRS) pain scores. **(B)** Changes in the frequency of daily breakthrough pain episodes. **(C)** Adjustments in morphine dosage over time.

Postoperatively, the patient’s pain improved dramatically. From postoperative day 0 to day 3, her NRS score decreased to 1-3, with only 1–2 episodes of breakthrough pain per day. During the 1- to 6-month follow-up, her pain control remained excellent (NRS 1-2), with virtually no breakthrough pain. Her quality of life and functional activity improved significantly, and no major complications related to the pump or catheter were observed. The trajectory of pain relief is summarized in **Figure 3**.

## 4 Discussion

This case report demonstrates that endoscopic assistance is a feasible and effective technique for intrathecal catheter placement in patients where standard percutaneous puncture fails due to complex altered spinal anatomy. The primary value of this approach lies in its ability to provide direct visual guidance, thereby converting a blind, potentially risky procedure into a precise and controlled one, and potentially obviating the need for conversion to open surgical implantation.

The failure of standard fluoroscopy-guided puncture in this patient was anticipated based on preoperative MRI findings of severe epidural fibrosis and obscured anatomical landmarks—common sequelae of previous surgeries, radiotherapy, or metastatic disease itself (7, 8). In such scenarios, repeated blind attempts not only increase procedural time and radiation exposure but also elevate the risks of dural injury, cerebrospinal fluid leak, and catheter malposition (9, 10). The endoscopic-assisted technique directly addresses this challenge. By providing real-time, magnified visualization of the ligamentum flavum, dura mater, and intrathecal space, it allows for precise needle entry and confirms catheter placement within the CSF, significantly enhancing procedural accuracy and safety (11, 12). This technique should therefore be considered as a planned rescue or even primary strategy for IDDS implantation in patients with predicted difficult anatomy, such as those with prior extensive spinal surgery, post-radiation fibrosis, or significant deformity.

A critical decision point after failed percutaneous puncture is whether to convert to an open laminectomy for catheter placement. This decision carries significant implications, as robust evidence from spine surgery demonstrates that open laminectomy, compared to minimally invasive techniques, is consistently associated with greater intraoperative blood loss, longer hospital stays, and higher reoperation rates (13). Such increased perioperative burden is particularly undesirable in frail patients with advanced cancer. The endoscopic-assisted technique presented here provides a minimally invasive intermediary solution. It preserves the core advantages of minimal access surgery while granting the direct visualization needed to overcome complex anatomical barriers, thereby effectively avoiding the need for conversion to a more invasive open procedure. While advantageous, this technique is not without demands. It requires specialized endoscopic equipment and, more importantly, a surgeon skilled in both endoscopic spinal surgery and the principles of intrathecal access. The learning curve involves transitioning from tactile/fluoroscopic feedback to visual-spatial navigation within a narrow corridor. However, for centers already performing spinal endoscopy for decompression or discectomy, the skills and equipment are largely transferable. The additional procedural time observed in this initial case is expected to decrease with experience and should be weighed against the time and risk of multiple failed punctures or a conversion to open surgery.

This report is limited by its nature as a single case. The efficacy and safety of this technique require validation in larger, prospective series. Furthermore, the cost of endoscopic equipment may limit its availability in some settings, though this may be offset by reducing costs associated with complications or repeat procedures. Future research should directly compare outcomes (success rate, complication rate, procedure time) of endoscopic-assisted versus conventional fluoroscopic-guided implantation in a cohort of patients with complex spinal anatomy. Additionally, standardized protocols for patient selection, endoscopic setup, and procedural steps need to be developed and disseminated.

In conclusion, endoscopic-assisted intrathecal catheter placement represents a valuable technical advancement for implanting IDDS in patients with refractory cancer pain and challenging spinal anatomy. It enhances procedural precision and safety, provides a viable minimally invasive alternative when standard puncture fails, and can prevent the need for more invasive open surgery. As the population of cancer survivors with complex treatment histories grows, this technique warrants further exploration and integration into the armamentarium of pain interventionalists.

## Data Availability

The original contributions presented in the study are included in the article/supplementary material. Further inquiries can be directed to the corresponding author.
